# Proposed Diploma for Irish Nurses

**Published:** 1917-05-05

**Authors:** 


### THE EDITOR'S LETTER-BOX.

### PROPOSED DIPLOMA FOR IRISH NURSES.

DECISION OF THE ROYAL COLLEGE OF SURGEONS, IRELAND.

*To the Editor of* The Hospital.

Sir,—The Irish Royal College of Surgeons have decided by resolution to carry through the scheme rejected by the Royal College of Physicians of granting diplomas to Irish nurses. This is, of course, a counter-proposition to the Royal British College of Nursing now established here. In itself, it is a harmless scheme. That is to say, a nurse who holds such a diploma will be no worse off on that account. But, all the same, the unthinking nurse may be deceived thereby, and I desire, with your permission, to put her on her guard. The regulations are not yet complete, but they are shortly to be promulgated. When they are, let the nurse first count the cost. If the diploma is to be issued free of charge, my first objection is removed. If not, she is paying for that which profiteth not. Her College of Surgeons' diploma will count for exactly as much as her present certificate of training in Ireland, and no more. Neither will it count for anything more outside of Ireland, and I am of opinion that already the Irish nurse has paid dearly enough for a certificate of her training. My second objection is that if the College of Surgeons' diploma can be obtained in Irish training-schools too small for recognition by the Royal British College of Nursing, the value of the diploma will at once automatically grade itself on a lower level. That is to say, if Training School A is accepted by both, and Training School B by the Royal College of Surgeons only, obviously the value of the Surgeons' diploma is inferior, even if the holder thereof trained at Training School A.

Of course, it is obvious to all that the Royal College of Surgeons' diploma cannot be of any value outside of Ireland.—Yours, etc., IERNE.

### Children's Country Holidays Fund.

*To the Editor of* The Hospital.

